# A call to join a collective effort on AI evaluation

**DOI:** 10.1016/j.patter.2026.101512

**Published:** 2026-03-06

**Authors:** Irving Torres, the Evals-Consensus.AI Consortium

**Affiliations:** 1 Evals-Consensus.AI Coalition, San Bruno, CA, United States

## Abstract

AI evaluations increasingly shape deployment, governance, and trust, but expectations for how they are conducted and reported remain fragmented. We introduce a cross-sector Delphi process to develop community-endorsed guidance for AI evaluation practice and invite researchers, practitioners, ethics organizations, and institutions to participate.

## Main text

AI evaluations now influence decisions far beyond research benchmarks, including deployment choices, objectives, regulatory scrutiny, procurement, and public trust. Yet despite their growing importance, there is little shared agreement on what constitutes a *well-designed* or *well-reported* evaluation. Practices vary widely, comparisons are difficult, and many practices that would address this are not adopted.^1^^,^^2^^,^^3^^,^^4^^,^^5^

This is not a failure of effort or expertise. On the contrary, the AI evaluation community has produced a large and growing body of thoughtful work, including frameworks, checklists, audits, and critiques, that could be adopted. The problem is that without a shared reference point, no single approach becomes the standard, creating a fragmented ecosystem.

Therefore, we invite participation by individuals and organizations across academic and ethics research, evaluation development, auditing, industry practice, and the public sector to develop shared AI evaluation expectations. This invitation marks the launch of a cross-sector Delphi study. Individuals may participate directly in the Delphi study, including by rating items, suggesting refinements, endorsing the final high-level statement, or assisting with synthesis and reporting.^6^ Organizations may participate by nominating contributors to the study and by endorsing the Delphi process, the resulting high-level statement, or both, signaling support for the legitimacy and integrity of the process. Participation does not require agreement with any particular view, only a willingness to state one clearly. This distinction is not new and has been essential in other fields facing similar problems.

Other domains facing similar coordination problems (medicine, law, public health, and evidence synthesis) have converged on a common response: structured, transparent consensus processes that distinguish agreement from disagreement rather than papering over it.^7^^,^^8^^,^^9^ Inspired by these precedents, the Delphi study is designed to surface where experts and practitioners agree about AI evaluation practice, where agreement is conditional, and where disagreement persists. Following the lead of these other projects, we also expect that the process will require routine updates, especially given the fast pace of AI development and emerging practices.

The project’s goal is deliberately limited: to produce a concise, non-binding checklist describing the recommended practices for conducting and reporting AI capability evaluations. The checklist is intended as guidance, not a compliance regime; its value lies in clarifying expectations, not enforcing uniformity. Equally important, areas without consensus will be explicitly marked rather than resolved by fiat.

The process leading to the initial checklist is fully preregistered, including participant stratification, rating scales, consensus thresholds, and reporting rules, and all details are publicly available.^6^ Participants will provide anonymous judgments over multiple rounds with controlled feedback between rounds. At the end, a detailed transparent record of expert judgments along with the materials, aggregation logic, and deviations from plan will be released. This design aims to reduce dominance effects, surface minority views, promote transparency, and make disagreement, whether normative or otherwise, legible.

AI evaluation will remain a value-laden, evolving practice. This effort does not pretend to settle that complexity. Instead, it replaces implicit assumptions with explicit, inspectable consensus by giving the field a clearer starting point for disagreement, improvement, and revision.

To express interest in participating in the initiative, please visit evals-consensus.ai and click “Participate!” to fill-out the form. The Delphi study is already actively recruiting, and the first round of Delphi inputs will begin before the end of March. While participants can join at any time during the Delphi process, we invite people to apply as soon as possible to have their views incorporated.

That is how humans have always met hard problems: not through decree or certainty, but by finding common ground that is imperfect, contested, and revisable and that helps us navigate complexity together.

## Consortia

Endorsing members of project consortium currently include AI Standards Lab, Apollo Research, FAR.AI, MIT AI Risk Initiative, and Purdue GRAIL. This is open to additional organizations, and such organizational endorsement does not imply agreement with the future results; it is instead an endorsement of the importance, legitimacy, and integrity of the process.

## Acknowledgments

Parts of the overall project work were contributed to or led by David Manheim, Andrea Loehr, Daniel S. Schiff, Michael Noetel, and Stephen Casper. Financial supporters of the work include Coefficient Giving (Open Philanthropy). We are also grateful for methodological and subject matter input from Patricia Paskov, Rishi Bommasani, and Olawale Salaudeen. This is a partial list, and endorsing and participant organizations and groups will expand through invitations and recruitment per the preregistered Delphi protocol.

## Declaration of interests

The coalition is comprised of members with a wide variety of financial, legal, or policy interests in AI evaluations and AI systems more generally, including constructing AI models, offering AI services, and having financial interests in evaluations and audit of AI systems. This diversity and inclusion of these stakeholders creates a wide variety of actual conflicts of interest about the topic, making a transparent and open process even more important.

## Declaration of generative AI and AI-assisted technologies in the writing process

During the preparation of this work, the author used GPT-5.2 to clarify and tighten language to improve readability. After using this tool, the author reviewed and edited the content as needed and takes full responsibility for the content of the publication.
